# Do hypoxia/normoxia culturing conditions change the neuroregulatory profile of Wharton Jelly mesenchymal stem cell secretome?

**DOI:** 10.1186/s13287-015-0124-z

**Published:** 2015-07-24

**Authors:** Fábio G. Teixeira, Krishna M. Panchalingam, Sandra Isabel Anjo, Bruno Manadas, Ricardo Pereira, Nuno Sousa, António J. Salgado, Leo A. Behie

**Affiliations:** Life and Health Sciences Research Institute (ICVS), School of Health Sciences, University of Minho, Braga, Portugal; ICVS/3B’s, PT Government Associate Laboratory, Braga/Guimarães, Portugal; Pharmaceutical Production Research Facility (PPRF), Schulich School of Engineering, University of Calgary, Calgary, AB Canada; CNC - Center for Neuroscience and Cell Biology, University of Coimbra, Coimbra, Portugal; Faculty of Sciences and Technology, University of Coimbra, Coimbra, Portugal; Biocant - Biotechnology Innovation Center, Cantanhede, Portugal

## Abstract

**Introduction:**

The use of human umbilical cord Wharton Jelly-derived mesenchymal stem cells (hWJ-MSCs) has been considered a new potential source for future safe applications in regenerative medicine. Indeed, the application of hWJ-MSCs into different animal models of disease, including those from the central nervous system, has shown remarkable therapeutic benefits mostly associated with their secretome. Conventionally, hWJ-MSCs are cultured and characterized under normoxic conditions (21 % oxygen tension), although the oxygen levels within tissues are typically much lower (hypoxic) than these standard culture conditions. Therefore, oxygen tension represents an important environmental factor that may affect the performance of mesenchymal stem cells *in vivo*. However, the impact of hypoxic conditions on distinct mesenchymal stem cell characteristics, such as the secretome, still remains unclear.

**Methods:**

In the present study, we have examined the effects of normoxic (21 % O_2_) and hypoxic (5 % O_2_) conditions on the hWJ-MSC secretome. Subsequently, we address the impact of the distinct secretome in the neuronal cell survival and differentiation of human neural progenitor cells.

**Results:**

The present data indicate that the hWJ-MSC secretome collected from normoxic and hypoxic conditions displayed similar effects in supporting neuronal differentiation of human neural progenitor cells in vitro. However, proteomic analysis revealed that the use of hypoxic preconditioning led to the upregulation of several proteins within the hWJ-MSC secretome.

**Conclusions:**

Our results suggest that the optimization of parameters such as hypoxia may lead to the development of strategies that enhance the therapeutic effects of the secretome for future regenerative medicine studies and applications.

## Introduction

The use of adult stem cells, including human mesenchymal stem cells (hMSCs), as a possible therapeutic tool in the regenerative medicine field has been widely assessed [1–3], and has emerged as a promising therapeutic approach [4–6]. The stem/progenitor cells present in the human Wharton Jelly of the umbilical cord, known as human Wharton Jelly mesenchymal stem cells (hWJ-MSCs), have been suggested as a possible population of interest for future clinical applications [7–9]. Like bone marrow MSCs or adipose stem cells, these populations are also defined as MSCs [10–12]. The secretion of trophic bioactive molecules (i.e. MSC secretome) has now been considered as a critical element for their therapeutic efficacy [13–16]. Indeed, over the last decade, there has been a substantial effort to assess the impact of MSCs (including hWJ-MSCs) and their secretome into different disorders, such as those affecting the central nervous system (CNS) [14]. However, despite promising results for hMSCs and their paracrine activity, the low number of cells that are normally obtained after isolation continues to be a limitation for their application in clinical conditions [17–19]. One promising strategy to overcome this limitation is to focus on the modulation of culture conditions in which the dissolved oxygen concentration may play an important role in the behavior of MSCs [17].

Usually, hMSCs are cultured *in vitro* in static culture and in a 21 % oxygen tension environment. However, studies have demonstrated that the physiological niches from where hMSCs are isolated in the human body are at much lower oxygen tensions than 21 % [20–22]. Indeed, depending of the environmental niche from where MSCs are isolated, oxygen tension can vary between 1 and 7 % in the bone marrow, and between 10 and 15 % in the adipose tissue [23–25]. Regarding birth-associated tissues such as the umbilical cord, the oxygen tension within the mammalian female reproductive tract was shown to be low, between 1.5 and 8 %, and lasts throughout the fetal development with a dissolved oxygen tension in the fetal circulation rarely exceeding 5 % [26, 27]. Even though consensus values of 3 to 5 % of oxygen in tissues are generally accepted, the actual oxygen concentration in situ strongly depends on the vascularization of the tissue and its metabolic activity [28, 29]. In line with this, studies have shown that hypoxic culture conditions affect the therapeutic properties of hMSCs [30, 31]. For instance, Rhijn and colleagues [17] demonstrated that hypoxic preconditioning enhances the regenerative potential of MSCs, maintaining their immunosuppressive capacities under these conditions. In addition, Tsai and colleagues [30] demonstrated that the use of 1 % oxygen reduces hMSC senescence while it increases their proliferation levels and maintains their differentiation properties. Similar outcomes were also described for hMSCs obtained from adipose tissue and Wharton Jelly [20, 32, 33]. Furthermore, in the secretome the oxygen tension seems to play an important role [34, 35]. Previous studies have shown that by changing the oxygen concentration it was possible to modulate the angiogenic potential of MSCs through the increase in the secretion of vascular endothelial growth factor (VEGF), beta-fibroblast growth factor (bFGF) and hepatocyte growth factor (HGF) [34–36]. Regarding hypoxic conditions, Volkmer and colleagues [37] observed that prolonged exposure to hypoxia leads to cell death. On the other hand, under normoxic conditions, studies have shown that higher levels of oxygen could be toxic, causing oxidative stress due to the generation of reactive oxygen species (ROS) which could alter the metabolic efficiency of the cells [21, 29]. Nevertheless, the real impact of oxygen on key hMSC characteristics is still unclear. Additionally, it has been shown that hMSCs respond to changes in their physiological environment [38], namely by using dynamic culturing environments such as those provided by bioreactors [38–40]. Indeed, previous work from our group demonstrated that, using stirred suspension bioreactors, a number of advantages can be achieved including: (1) a large number of cells can be expanded in one vessel (minimizing vessel-to-vessel variability and minimizing cost related to labor and consumables); (2) the bioreactors can be operated in a fed-batch or perfusion mode of operation; and (3) the bioreactors can be set up with computer-controlled online monitoring instruments to ensure tight control of process variables such as pH, temperature and dissolved oxygen concentration.

Thus, in the present work we aimed to characterize and analyze the effects of the hWJ-MSC secretome collected from hypoxic culture conditions and compare that to those obtained from normoxic culturing conditions. Results revealed that the use of different oxygen conditions (i.e., hypoxic and normoxic) led to a different secretome profile for hWJ-MSCs. In line with this, we further observed that hWJ-MSCs were able to secrete important neuroregulatory molecules such as glia-derived nexin (GDN) and cystatin C (Cys C), which were upregulated under the normoxic condition. In the hypoxic condition, the proteins thymosin-beta, elongation factor 2 (EF-2), ubiquitin carboxy-terminal hydrolase L1 (UCHL1), clusterin, peroxiredoxin-1 (Prx1) and 14-3-3, were found to be upregulated in the hWJ-MSC secretome. Additionally, we have also found vitronectin, cadherin-2 and multidrug resistance-associated protein 1 (MRP1) were expressed only in the normoxic conditions, while pigment epithelium-derived factor (PEDF), insulin growth factor 2 (IGF-2), semaphorin-7A, macrophage migration inhibitory factor (MIF), heat shock protein 70 kDa (Hsp70) and moesin were only found to be present in the hypoxic conditions. Finally, we also observed that the obtained secretomes were able to induce and support neuronal differentiation of human telencephalon neural precursor cells (hNPCs) *in vitro*, where the previously identified proteins in the hWJ-MSC conditioned medium (CM) may explain our results.

## Methods

### Expansion of hWJ-MSCs under hypoxic and normoxic conditions (in dynamic bioreactors) and collection of CM

#### Preparation of 500 mL suspension bioreactors

A DASGIP Parallel Bioreactor system (DASGIP, Julich, Germany) was used for the expansion of hWJ-MSCs in dynamic conditions. Prior to inoculating hWJ-MSCs in the DASGIP bioreactors, the 500 mL suspension bioreactors (containing modified Teflon 4-paddle impellers) were siliconized using Sigmacote (Sigma, St. Louis, MO, USA) to minimize cell attachment to the sides of the bioreactor vessel and the impeller. After siliconization and autoclaving of the vessels, the DASGIP system was calibrated according to procedures provided by the manufacturer. The bioreactors were maintained at: (1) 37 °C using a heating jacket; (2) 100 % dissolved oxygen (corresponding to oxygen saturation of the medium at 37 °C exposed to 21 % O_2_ in the headspace) for normoxic conditions; (3) 21 % dissolved oxygen (corresponding to oxygen saturation of the medium at 37 °C exposed to 5 % O_2_ in the headspace) for hypoxic conditions; (4) pH of 7.4, controlled by a gas mixture connected to oxygen, nitrogen, carbon dioxide and air tanks that was introduced into the headspace; and (5) agitation of 52 rpm using a magnetic stir plate under the bioreactors.

#### Preparation of microcarriers and inoculation of hWJ-MSCs

Cytodex 3 microcarriers (GE Healthcare, Uppsala, Sweden) were used for this study and were prepared as follows: 1.0 g (per condition) of microcarriers were weighed out and hydrated in 50 mL 1×phosphate-buffered saline (PBS; Life Technologies, Paisley, UK) in two 125 mL, pre-siliconized, Erlenmeyer flasks, at room temperature overnight. To this flask, 2–3 drops of Tween 80 (United States Biochemical Corporation, Cleveland, OH, USA) were added to break the surface tension and ensure proper wetting and sedimentation of the microcarriers. The microcarriers were then washed three time with 1×PBS, and autoclaved. Following autoclaving, the microcarriers were incubated with fetal bovine serum (FBS; Life Technologies, Paisley, UK) for 6 h at 37 °C to coat the microcarriers with serum-attachment factors, and agitated every 30 min. After 6 h, the FBS was removed, the microcarriers were washed twice with our serum-free medium (PPRF-msc6), and then inoculated into our 500 mL DASGIP bioreactors in 275 mL of serum-free medium for 4 h at the controlled culture conditions. The hWJ-MSCs were first expanded in Nunc T-flasks (Thermo Scientific, Waltham, MA, USA) pre-coated with a 0.1 % gelatin solution. The gelatin solution was prepared by adding 0.1 g of Type B bovine gelatin (Sigma, St. Louis, MO, USA) to 100 mL of cell culture water (Lonza, Walkersville, MD, USA). This was then autoclaved to both sterilize the solution and dissolve the gelatin. After autoclaving, the gelatin was then cooled in the 4 °C fridge overnight. T75 T-flasks were coated with gelatin by: 1) adding 4 mL of gelatin to the flask and letting it sit for 30 min, 2) then removing the gelatin from the flask, and 3) allowing the flask to dry overnight before use. After this, cryopreserved hWJ-MSCs at passage 2 (P2), and derived from three donors (WJ1, WJ2, and WJ3) were expanded in serum-free medium (PPRF-msc6 [41]) in static culture for two passages before inoculation into the DASGIP bioreactors. The cells were harvested using trypsin-EDTA, and then inoculated into the bioreactors at a density of 24,000 cells/mL (based on the final volume of 500 mL) and the volume of the bioreactors was maintained at 325 mL for the first 24 h to increase cell attachment. After 24 h the culture volume was increased to 500 mL to bring the final microcarrier density to 2.0 g/L. The cells were cultured on the microcarriers for 72 h, after which time the bioreactors were removed from the DASGIP system, and placed in a biosafety cabinet for 10 min to allow the microcarriers to settle. The supernatant was removed from the bioreactors, and the microcarriers were washed once with 100 mL of Neurobasal®-A medium (Life Technologies). Following this, 500 mL of Neurobasal®-A medium with 1 % of kanamycin (Life Technologies) were added to the bioreactors and the bioreactors were placed back into the DASGIP control system for 24 h. After 24 h, the bioreactors were again removed from the system, placed in a biosafety cabinet for 10 min to allow the microcarriers to settle, the supernatant was harvested and centrifuged at 300 g for 10 min to remove any cell debris. This supernatant (called the dynamic CM) collected from normoxic and hypoxic conditions was then stored at −80 °C until it was required for further experiments.

### Growth of hNPCs and incubation with hWJ-MSC CM collected from normoxic and hypoxic conditions

hNPCs were isolated from the telencephalon region of a 10-week postconception (gestational age) fetus using the protocols developed at the Queen Elizabeth II Health Sciences Centre under strict ethical guidelines [42–44]; ethical consent was approved by the Conjoint Health Research Ethics Board (CHREB), University of Calgary (ID: E-18786). Pre-isolated and cryopreserved hNPCs were thawed at 37 °C and the contents placed into a Nunc T-25 flask (Thermo Scientific) containing 5 mL of a serum-free medium PPRF-h2 [42]. After 2 days, the cells were harvested and mechanically dissociated into a single cell suspension, and subcultured into fresh cell growth medium (PPRF-h2). Every 4 days, the T flasks were fed by replacing 40 % of the spent medium with fresh growth medium. After 14–20 days of growth in the culture flasks, hNPCs were passaged and plated onto pre-coated (poly-D-lysine hydrobromide (100 μg/mL) and laminin (10 μg/mL); Sigma) 24-well plates at a density of 4.0×10^4^ cells per well in the presence of the hWJ-MSC CM obtained from either hypoxic or normoxic conditions. Neurobasal-A medium (Life Technologies) with 1 % of kanamycin (Life Technologies) was used as control, without the addition of any kind of exogenous cocktail of factors, as already described by our research group [45, 46]. The plates were placed in an incubator operating at 37 °C, 5 % CO_2_, 95 % air and 90 % relative humidity for 5 days.

### Immunostaining

hNPCs were fixed in 4 % paraformaldehyde (Mallinckrodt, Paris, KY, USA) for 15 min, and then permeabilized by incubation with 0.1 % Triton X-100 (Sigma) in 1×PBS for 5 min at room temperature, and washed three times in 1×PBS. hNPCs were then blocked with 10 % fetal calf serum (FCS; Life Technologies) in 1×PBS, followed by a 1-h incubation (at 37 °C) with the primary antibodies: rabbit anti-doublecortin (DCX; 1:500, Abcam, Cambridge, MA, USA) to detect immature neurons and mouse anti-rat microtubule associated protein-2 (MAP-2; 1:500, Sigma) to detect mature neurons. hNPCs were then washed with 1×PBS three times and incubated with the secondary antibodies: Alexa Fluor 488 goat anti-rabbit (Life Technologies) and Alexa Fluor 594 goat anti-mouse immunoglobulin G (IgG; Life Technologies) for 1.0 h at 37 °C and then 10 min with 4-6-diamidino-2-phenylindole-dihydrochloride (DAPI; Life Technologies). After staining, samples were observed under an Olympus BX-61 Fluorescence Microscope (Olympus, Hamburg, Germany). For quantification purposes, cell counts were performed under blind observation by using Cell-P software (Olympus). For this, three cover slips and ten representative fields per condition were chosen and analyzed. In order to normalize the data between the different sets, the results are presented in percentage of cells. This was calculated by counting the cells positive for MAP-2/DCX markers and dividing this value by the total number of cells/field (DAPI-positive cells; n = 3).

### Proteomics – mass spectrometry and SWATH acquisition

#### Liquid digestion/sample preparation

hWJ-MSC CM was firstly concentrated using a Vivaspin 20 sample concentrator (5 kDa; GE Healthcare) by ultracentrifugation at 3000 g for 45 min. Then, the CM was precipitated with Trichloroacetic acid (TCA)–acetone. TCA was added to each sample to a final concentration of 20 % (v/v), followed by 30 min incubation at −80 °C and centrifugation at 20,000 *g* for 20 min. Protein pellets were washed with ice-cold (−20 °C) acetone, briefly the pellets were solubilised in acetone, aided by ultrasonication, followed by a centrifugation at 20,000 *g* for 20 min. The washed pellets were resuspended in 1.0 M triethylammonium bicarbonate buffer (TEAB; Sigma), aided by ultrasonication, followed by a centrifugation at 20,000 *g* for 5.0 min to remove insoluble material [47].

Samples were quantified using the 2D-Quant Kit (GE Healthcare) and 100 μg of each sample were subjected to liquid digestion. Briefly, 4 μL of 50 mM tris (2-carboxyethyl)phosphine (TCEP) was added to 45 μL of sample, followed by an ultrasonication step for 2 min. Next, 2 μL of 600 mM methyl methathiosulfonate (MMTS) was added and samples were left to react for 10 min at room temperature. TEAB was then added to adjust the volume of sample to 100 μL, and the samples were digested with trypsin overnight (2 μg trypsin/sample) at 37 °C, with swirling at 650 rpm. Reactions were stopped by the addition of 2 μL formic acid (FA) and the peptides were dried by rotary evaporation under vacuum. Before digestion, the samples were spiked with 2 μg green fluorescent protein (GFP) to monitor samples loss during the procedure.

Before performing the tandem mass spectrometry (MS/MS) analysis the peptide mixtures were cleaned/desalted using OMIX tips with C18 stationary phase (Agilent Technologies, Santa Clara, CA, USA) as recommended by the manufacturer. Eluates, spiked with iRT peptides (Biognosys, Zürich, Switzerland), were dried by rotator evaporation, avoiding totally evaporating the samples. The samples were resuspended to 23 μL in a solution of 2 % acetonitrile (ACN) and 0.1 % FA followed by vortex, spin and sonication in a water bath for 2 min with pulses of 1.0 s (1.0 s sonication followed by a 1.0 s break pulse), at 20 % intensity, in a sonicator VibraCell 750 W, Sonics® (Sonics&Materials, Stowmarket, UK). In order to remove insoluble material the peptide mixture was then centrifuged for 5 min at 14,000 g and collected into the proper vial for liquid chromatography-mass spectrometry (LC/MS) injection.

#### SWATH acquisition

Samples were analyzed on a Triple TOF™ 5600 System (ABSciex, Framingham, MA, USA) in two phases: information-dependent acquisition (IDA) was followed by SWATH (Sequential Windowed data independent Acquisition of the Total High-resolution Mass Spectra) acquisition on the same sample [48]. Peptides were resolved by liquid chromatography (nanoLC Ultra 2D, Eksigent, California, USA) on a ChromXP™ C18AR reverse phase column (300 μm ID × 15 cm length, 3 μm particles, 120 Å pore size; Eksigent) at 5 μL/min. Peptides were eluted into the mass spectrometer with an ACN gradient in 0.1 % FA (2 % to 35 % ACN, in a linear gradient for 25 min), using an electrospray ionization source (DuoSpray™ Source, ABSciex).

For IDA, the mass spectrometer was set to scanning full spectra (350–1250 m/z) for 250 ms, followed by up to 30 MS/MS scans (100–1500 m/z for 75 ms each). Candidate ions with a charge state between +2 and +5 and counts above a minimum threshold of 70 counts/s were isolated for fragmentation and one MS/MS spectra was collected for 75 ms before adding those ions to the exclusion list for 15 s (mass spectrometer operated by Analyst® TF 1.6, ABSciex). Rolling collision was used with a collision energy spread of 5. Peptide identification was performed with Protein Pilot software (v4.5, ABSciex). Search parameters used were the following: SwissProt database, against a database composed of human and bovine species from SwissProt database (release at February 2014), GFP and iRT peptides, and using MMTS alkylated cysteines as fixed modification. An independent false discovery rate (FDR) analysis using the target-decoy approach provided with the Protein Pilot software was used to assess the quality of the identifications, and positive identifications were considered when identified proteins and peptides reached a 5 % local FDR [49, 50].

The SWATH setup was essentially that used by Gillet et al. [51], with the same chromatographic conditions used as in the IDA run described above. For SWATH-MS based experiments, the mass spectrometer was operated in a looped product ion mode. The instrument was specifically tuned to allow a quadruple resolution of 25 m/z mass selection. Using an isolation width of 26 m/z (containing 1 m/z for the window overlap), a set of 30 overlapping windows was constructed covering the precursor mass range of 350–1100 m/z. A 250 ms survey scan (350–1500 m/z) was acquired at the beginning of each cycle for instrument calibration and SWATH MS/MS spectra were collected from 100–1500 m/z for 90 ms resulting in a cycle time of 3 s from the precursors ranging from 350 to 1100 m/z. The collision energy for each window was determined according to the calculation for a charge +2 ion centered upon the window with a collision energy spread of 15.

A specific library of precursor masses and fragment ions was created by combining all files from the IDA experiments, and used for subsequent SWATH processing. Libraries were obtained using Protein Pilot™ software (v4.5, ABSciex) with the same parameters as described above. Data processing was performed using SWATH™ processing plug-in for PeakView™ (v2.0.01, ABSciex); briefly peptides were selected automatically from the library using the following criteria: (1) the unique peptides for a specific targeted protein were ranked by the intensity of the precursor ion from the IDA analysis as estimated by the ProteinPilot™ software; and (2) peptides that contained biological modifications and/or were shared between different protein entries/isoforms were excluded from selection. Up to 15 peptides were chosen per protein, and SWATH™ quantitation was attempted for all proteins in the library file that were identified below 5 % local FDR from ProteinPilot™ searches. In SWATH™ Acquisition data, peptides are confirmed by finding and scoring peak groups, which are a set of fragment ions for the peptide. Target fragment ions, up to 5, were automatically selected and peak groups were scored following the criteria described in Lambert et al. [52]. Peak group confidence threshold was determined based on a FDR analysis using the target-decoy approach and 1 % extraction FDR threshold was used for all the analyses. Peptides that met the 1 % FDR threshold in one of the samples were retained, and the peak areas of the target fragment ions of those peptides were extracted across the experiments using an extracted-ion chromatogram (XIC) window of 3.0 min. The levels of the human proteins were estimated by summing all the transitions from all the peptides for a given protein (an adaptation of [53]) and normalized to the more stable internal standard.

### Statistical analysis

Statistical evaluation was performed using one-way analysis of variance and Student’s *t*-test through the program GraphPad Prism 5 (GraphPad Software Inc., La Jolla, CA, USA). Data are presented as mean ± SEM. The significance value was set at *p* < 0.05.

## Results

### Expansion of hWJ-MSCs under normoxic and hypoxic conditions on Cytodex 3 microcarriers in dynamic conditions

**Fig. 1:**
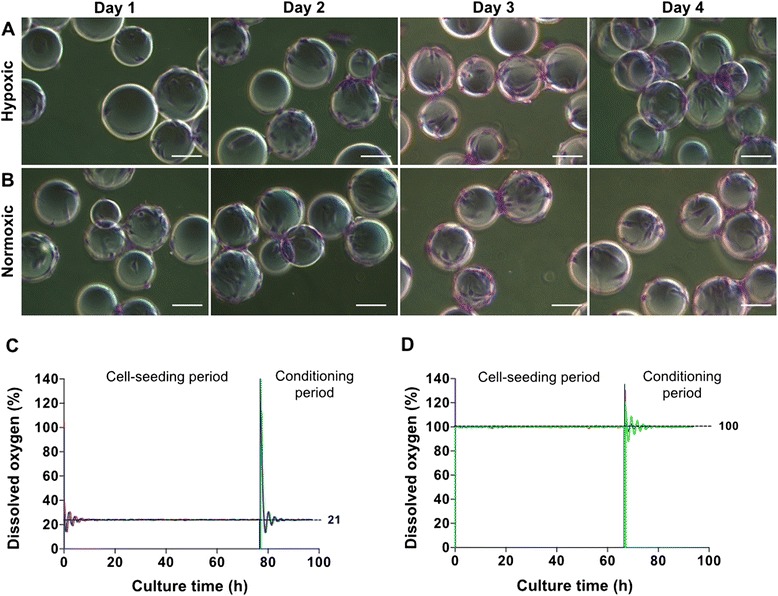
Expansion and adherence of hWJ-MSCs on Cytodex-3 microcarriers in computer-controlled bioreactors. hWJ-MSCs visualized by staining with 0.5 % crystal violet in methanol, were able to adhere to microcarriers under **a** hypoxic and **b** normoxic conditions in the suspension bioreactors, and were well maintained at predetermined set points for the culture period (**c**, **d**). Scale bars: 200 μm

PPRF-msc6 has been shown to support the rapid and efficient isolation and expansion of human bone marrow MSCs (hBM-MSCs) from bone marrow mononuclear cells. Additionally, higher cell yields were obtained in comparison to hBM-MSCs that were isolated and expanded in classical serum-based medium over the same culture period [19, 41]. Moreover, this serum-free medium was shown to support the isolation and expansion of hMSCs derived from adipose, pancreatic and umbilical cord in conventional static culture [54]. Herein, we report that hWJ-MSCs were able to attach to and grow on Cytodex 3 microcarriers in computer-controlled stirred suspension bioreactors in normoxic and hypoxic conditions (Fig. 1a, b). We also set and controlled key parameters in this dynamic environment and observed that dissolved oxygen (21 % (normoxic) and 5 % (hypoxic)) was well controlled within the bioreactor at the set points during the expansion and conditioning phase as illustrated in Fig. 1c, d. Cell viability analysis using a Vi-Cell XR Cell Viability Analyzer (Beckman Coulter, Danvers, MA, USA) revealed a percentage of viable cells above 98 %.

### Hypoxic and normoxic hWJ-MSC secretomes induced neuronal differentiation of hNPCs *in vitro*

**Fig. 2:**
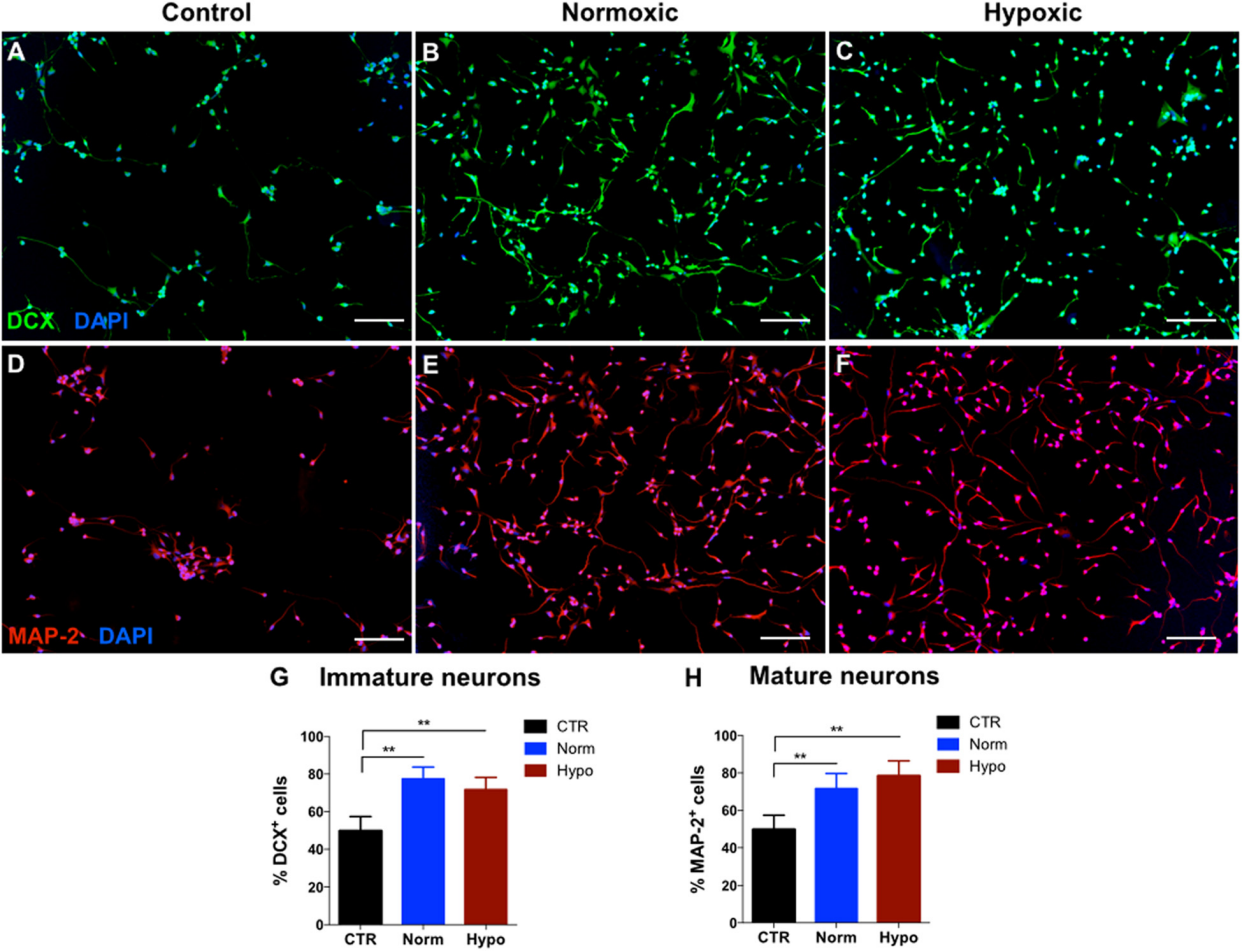
*In vitro* differentiation of hNPCs into immature and mature neurons. hWJ-MSC CM collected from hypoxic (Hypo) and normoxic (Norm) conditions was able to significantly support and induce neuronal differentiation of hNPCs into **b**,**c** immature (DCX^+^ cells) and **e**,**f** mature (Map-2^+^ cells) neurons when compared to control conditions (**a**,**d**). From **g**,**h** cell count analysis it was clearly evident the significant increase of DCX^+^ and Map-2^+^ cell densities in both hWJ-MSC CM (from normoxic and hypoxic conditions) when compared to the control group (values are shown as mean ± SEM. **p < 0.01). Scale bar: 100 μm. *CTR* control, *DAPI* 4-6-diamidino-2-phenylindole-dihydrochloride, *DCX* doublecortin, *MAP*-*2* microtubule associated protein-2

hNPCs were grown as neurospheres in a serum-free medium (PPRF-h2) [42]. The impact of hWJ-MSC secretomes (from hypoxic and normoxic conditions) on the differentiation of hNPCs was evaluated. Upon the removal of PPRF-h2, and plating in adherent dishes with hWJ-MSC normoxic or hypoxic secretomes, hNPCs adhered and started to differentiate. After 5 days incubated with hWJ-MSC CM, a Vi-Cell XR Cell Viability Analyzer (Beckman Coulter) was used to verify that the percent of viable cells during the total time in the CM was maintained above 95 %. In terms of differentiation, immunocytochemistry analysis revealed that when hNPCs were incubated for 5 days with the hWJ-MSC CM of both conditions (normoxic and hypoxic) there was a clear increase (*p* < 0.01) in DCX^+^ positive- (immature neurons; Fig. 2b, c, g) and MAP-2 positive cell (mature neurons; Fig. 2e, f, h) densities when compared to the control group (incubation with Neurobasal-A medium; Fig. 2a, d), suggesting that both secretomes (from normoxic and hypoxic conditions) favored hNPC differentiation.

### Hypoxic and normoxic conditions affect the profile of hWJ-MSC secretome

**Fig. 3:**
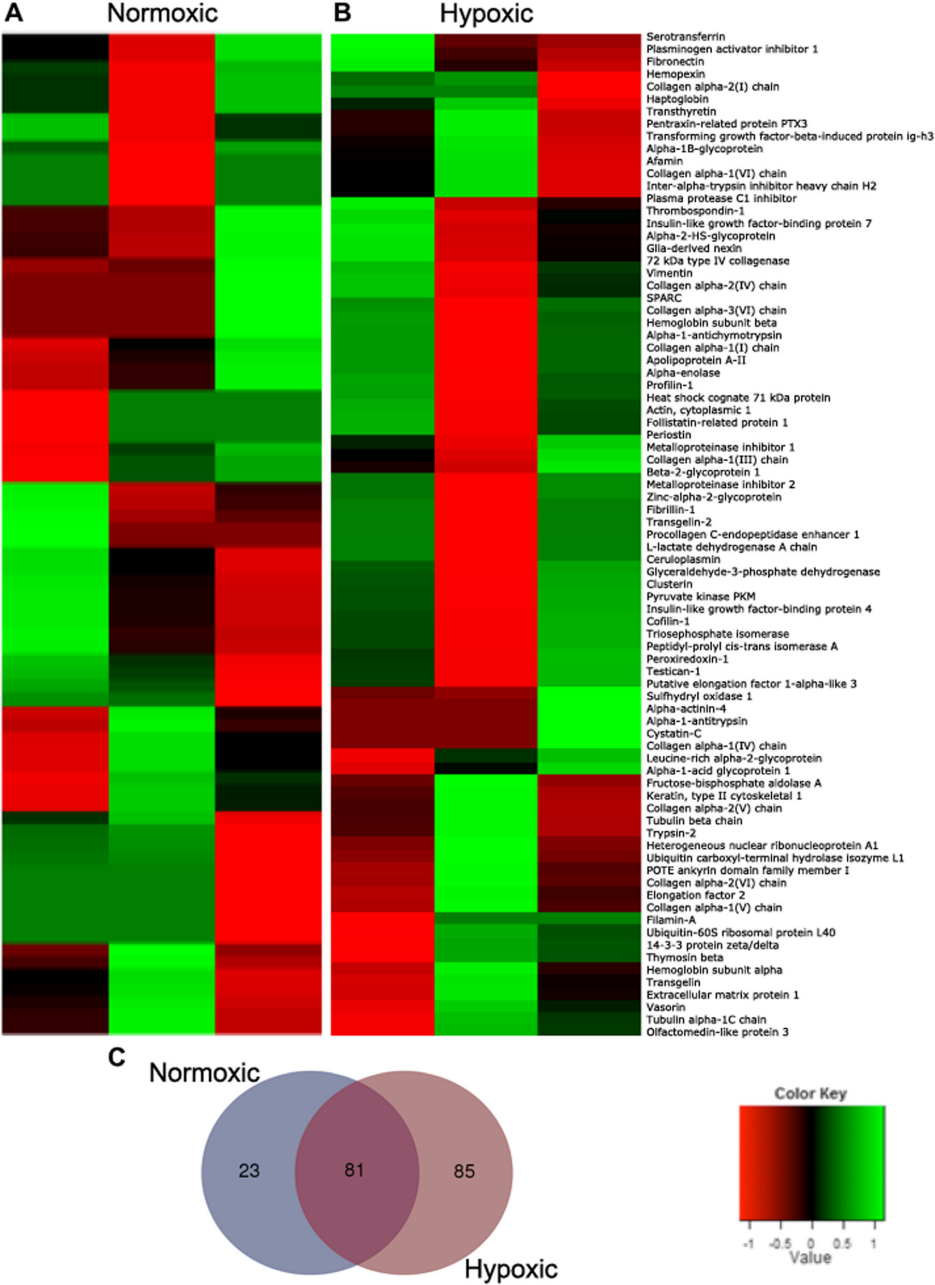
hWJ-MSC CM proteomic analysis. Graphical representation of WJ-MSC CM proteomic analysis by mass spectrometry. Peaks detected (from different donors: WJ1, WJ2, WJ3) after CM analysis show that the patterns of protein expression are modulated when we change from **a** normoxic to **b** hypoxic culture conditions. Indeed, the Venn diagrams (**c**) indicated that more proteins were identified in the hWJ-MSC CM collected from hypoxic conditions (166 proteins) when compared to the normoxic hWJ-MSC CM (104 proteins), in which 81 were common to the two conditions. The color scale shown illustrates the relative expression of the indicated proteins across the samples: red denotes low expression and green denotes high expression

**Fig. 4:**
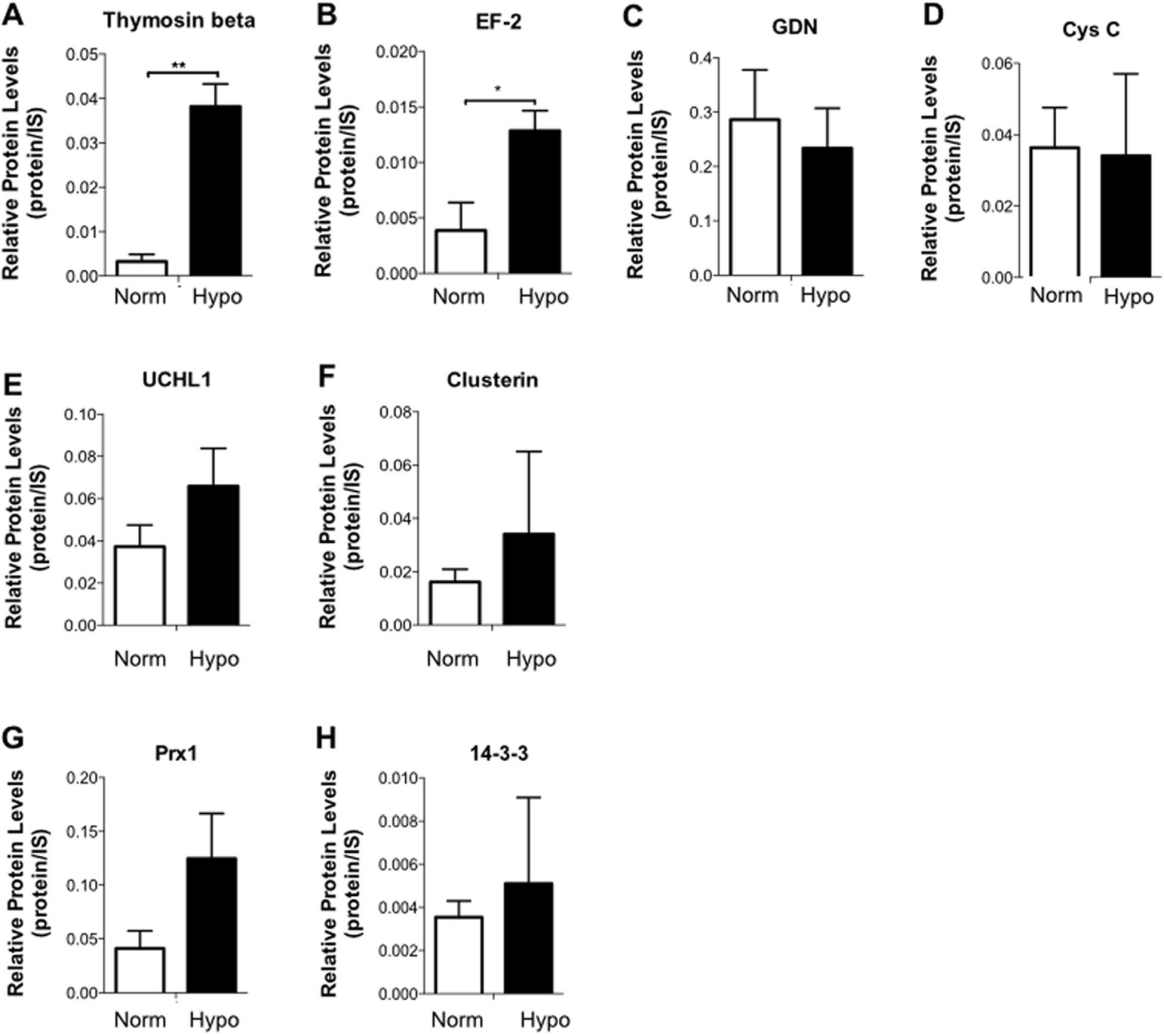
Specific hWJ-MSC CM proteins with neuroregulatory potential on CNS physiology. Comparative analysis of the secreted paracrine factors in the hWJ-MSC CM collected from hypoxic (Hypo: **A**- thymosin-beta; **B**- elongation factor 2 (EF-2); **E**- ubiquitin carboxy-terminal hydrolase L1 (UCHL1); **F**- clusterin; **G**- peroxiredoxin 1 (Prx1) and **H**- 14-3-3) and normoxic (Norm: **C**- glia-derived nexin (GDN); **D**- cystatin C (Cys C)) conditions with known neuregulatory actions. Values are shown as mean ± SEM, n = 3; *p < 0.05; **p < 0.01. *IS* internal standard

In order to further understand the effects of using different percentages of oxygen (i.e., normoxic and hypoxic conditions) on the secretome of hWJ-MSCs, a proteomic-based analysis was performed. From the results, it was observed that the use of different oxygen percentages modulated the hWJ-MSC secretome to produce a different pattern of expression (Fig. 3a, b), in which the hypoxic preconditioning led to an increased secretion profile of hWJ-MSCs when compared to the normoxic preconditioning. Indeed, it was possible to identify under normoxic conditions 104 proteins, whereas under hypoxic conditions 166 proteins were identified in which 81 proteins were common to the two conditions (Fig. 3c). After this proteomic analysis identification, when both hWJ-MSC secretomes were analyzed for specific proteins with possible neuroregulatory actions on the CNS physiology (including on their derived cells), it was possible to observe that hWJ-MSCs secreted important molecules. Thymosin-beta (*p* < 0.01) and EF-2 (*p* < 0.05) were found to be significantly upregulated in the hypoxic conditions when compared to the normoxic-related levels (Fig. 4a, b). On the other hand, for all the other proteins found (also with neuroregulatory potential), although without significant changes, positive trends between the two different conditions (normoxic and hypoxic) were observed. Regarding normoxic conditions, GDN and Cys C expression were found to be relatively upregulated when compared to the hypoxic conditions (Fig. 4c, d). Similar to this, but with a clear positive trend, UCHL1, clusterin, Prx1 and 14-3-3 were found to have higher secretion levels when compared to the normoxic conditions (Fig. 4e–h). In addition to this, specific proteins that were restricted to each condition were detected, presenting important roles in CNS regulation. Under normoxic conditions the presence of vitronectin, cadherin-2 and MRP-1 was identified whereas, under the hypoxic conditions, PEDF, IGF-2, semaphorin-7A, MIF, Hsp70 and moesin were identified.

## Discussion

Reports have defended the proposition that the stem cell niche represents and plays an important role in stem cell biology and fate, in which oxygen concentration is an important component and regulator [20, 55]. Indeed, it has been suggested that low levels of oxygen (i.e., hypoxia) play an important role in the maintenance of the plasticity and proliferation of stem cells [22]. According to Cicione et al. [21] and Grant et al. [56] the conventional in vitro cultures are often carried out under ambient oxygen tension corresponding to 21 %, called the normoxic condition. However, the in vivo physiologic oxygen concentration is lower than this, varying from tissue to tissue and ranging from 1 to 13 % [56]. In this way, the use of 21 % oxygen tension exceeds the normal pressure that exists in most of the mammalian tissues, which indicates that the oxygen concentration used during standard in vitro cultures of primary human cells, like MSCs, does not mimic the in vivo environment [21, 57]. Additionally, it has been hypothesized that culturing hMSCs under normoxic conditions could lead to a reduction in their therapeutic potential [58].

First, we showed that the in vitro application of the hWJ-MSC secretomes obtained from normoxic and hypoxic conditions revealed that both were able to maintain cell viability and induce differentiation of human CNS-derived cells. As seen in Fig. 2, when hNPCs were incubated with normoxic and hypoxic secretomes, similar levels of neuronal differentiation (namely, immature (DCX^+^ cells) and mature (Map-2^+^ cells) neurons), were observed. This is in line with what Sart and colleagues [59] have already reported regarding the effects of hypoxic and normoxic hMSC CM on NPC survival, differentiation and maturation. In addition, they also observe that both CM (from normoxic and hypoxic conditions) were also able to enhance the proliferation, migration and neurite outgrowth of NPCs [59]. Factors such as brain-derived neurotrophic factor (BDNF), nerve growth factor (NGF), fibroblast growth factor 2 (FGF-2), stem cell-derived factor 1 alpha (SDF-1α) and transforming growth factor beta 1 (TGF-β1) secreted by MSCs have been considered the major players involved in their potential for promoting growth and neural differentiation of hNPCs [13, 60–62]. Furthermore, it has been hypothesized that hMSC secretome could also be a stimulator of endogenous extracellular matrix (ECM) proteins secretion by NPCs, acting as a modulator of NPC behavior by influencing the ratio of adherence and differentiation [59]. This clearly suggests that the hMSC secretome may be able to enhance the neural survival and differentiation of NPCs due to the regulation of the molecular milieu, including ECM proteins and growth factors [59, 63, 64].

The proteomic analysis performed in the present work revealed that hWJ-MSCs were able to produce molecules with neuroregulatory potential other than those commonly described in the literature, particularly under hypoxic conditions. Of these, thymosin-beta and EF-2 were found to be significantly upregulated under hypoxic conditions when compared to the normoxic conditions (Fig. 4a, b). To a lesser extent, GDN and Cys C were found to be upregulated in normoxic conditions (Fig. 4c, d), whereas UCHL1, clusterin, Prx1 and 14-3-3 secretion tended to be higher under hypoxic conditions (Fig. 4e–h). Together, all these proteins have been reported to have important roles in neurite outgrowth, inhibition of apoptosis, neuroprotection, antioxidant activities and angiogenic effects [65–72]. For instance, thymosin-beta (significantly expressed, *p* < 0.01; Fig. 4a) and EF-2 (significantly expressed; *p* < 0.05; Fig. 4b) have been associated with important roles in the regulation of neurite outgrowth as well as neuroprotective actions in Alzheimer's disease [71–74]. GDN and Cys C (Fig. 4c, d) are known to play crucial roles in the enhancement of neurite outgrowth and neuroprotection through the prevention of oxidative stress [65, 75–78]. On the other hand, UCHL1 and clusterin (Fig. 4e, f) have been described as important enhancers of neuroprotection and neurogenesis (e.g., neuronal process formation, elongation, and plasticity) as well as a potential target into some neurodegenerative disorders such as Alzheimer’s disease [67, 79–82]. Finally, Prx1 and 14-3-3 (Fig. 4g, h) are known for their important roles on regeneration, cell migration, and axonal growth, as well as for neurite outgrowth and neuroprotection [45, 68, 69, 83, 84]. Interestingly, in addition to this, we have also discovered specific proteins that were only detected in each condition. From these, vitronectin, cadherin 2 and MRP1 (only present in the normoxic hWJ-MSC CM) have been described as important modulators of neuronal differentiation, axonal growth and neuroprotection [85–90]. On the other hand, PEDF, IGF-2, semaphorin-7A, MIF, Hsp70 and moesin (only present in the hypoxic hWJ-MSC CM) are known for their roles in the enhancement of neuroprotection, axonal growth, neurite outgrowth and neuronal cell survival and differentiation [90–99]. Interestingly, previous results from our group have identified the presence of some of these proteins, such as UCHL1, 14-3-3 and Hsp70, that were correlated with an increase in neuronal cell densities in cortical and cerebellar primary cultures [45].

Like Sart and colleagues [59], we have also observed that the hypoxic CM displayed similar cellular responses on hNPC differentiation when compared to normoxic conditions. This was surprising as the proteomic analysis revealed that there was a robust increase in most of the proteins referred to above for the secretome collected under hypoxic conditions. We believe that this could indicate that the differences in concentrations of the proteins that were detected may not be sufficient to induce significant variation in the functional differentiation of hNPCs. Similar outcomes were also presented by the other authors who observed that hypoxic conditions could increase the secretory profile of MSCs (increasing the levels of FGF-2, BDNF and VEGF) when compared to normoxic conditions. However, the dose/level obtained may not be sufficient to elicit differences in cell adhesion/proliferation/differentiation of NPCs when compared to the normoxic conditions [59]. The collection time of the hMSC secretome might be one of the reasons that could limit its effects on hNPC differentiation, as it has already been demonstrated that the time of conditioning affects the hMSC secretome protein profile even under hypoxic conditions [100, 101]. In addition to this, the oxygen tension could be another important issue. In fact, as recently demonstrated by Hsiao and colleagues [100], the paracrine profile of MSC-like cells may be dependent on the oxygen concentration. Nevertheless, it was evident that a dynamic culturing environment, such as the use of hypoxic conditions, leads to significant changes in the composition of the hWJ-MSC secretome. Although the mechanisms responsible for these changes are still not fully understood, studies have suggested that one of the mechanisms by which MSCs respond to oxygen tension is through transcription factors, namely the hypoxia-inducible factors HIF-1α and HIF-2α [20, 22]. These factors are degraded under normoxic conditions, but stabilized under hypoxic conditions, playing essential roles in a variety of cellular and molecular mechanisms of MSCs [21, 102, 103]. In addition to this, it was also demonstrated that proangiogenic factor production (e.g., IL-6 and VEGF) by MSCs was increased under hypoxic preconditioning, assuming that these beneficial effects might be also regulated by a complex array of signaling pathways such as the Akt and ERK pathways [104–107]. Taking all this into consideration, future standardized experiments should be developed in order to analyze the use of different hypoxic oxygen concentrations (for instance, below 5 %) and compared to the normoxic state, in order to get an insight of the real action of low levels of oxygen on the hMSC secretome physiology.

## Conclusions

In the present work, we have demonstrated that the secretomes of hWJ-MSCs collected from normoxic and hypoxic conditions were able to induce neuronal differentiation of hNPCs into neurons in different stages of maturation. These outcomes were associated with the presence of important neuroregulatory molecules within the constitution of the secretomes such as GDN, Cys C, UCHL1, clusterin, Prx1, 14-3-3, thymosin-beta and EF-2. These are important molecules involved in the promotion of neuroprotection, inhibition of apoptosis, angiogenesis and neuronal cell survival and differentiation. Thus, our results suggest that the use of hypoxic preconditioning enhances the hWJ-MSC secretome when compared to the normoxic precondition, indicating that hWJ-MSCs differ in their sensitivity when exposed to different oxygen concentrations, which may allow the development of new therapeutic strategies in the future. However, with conditions tested in the present study there were no changes in the neuroregulatory (neural differentiation) profile of the secretome. In the future, different hypoxia oxygen concentrations should be tested in order to identify the optimal parameters for enriching the WJ-MSC secretome under a dynamic and hypoxic environment.

## Abbreviations

ACN: Acetonitrile
BDNF: Brain derived neurotrophic factor
bFGF: beta-fibroblast growth factor
CM: Conditioned medium
CNS: Central nervous system
Cys C: Cystatin C
DAPI: 4-6-diamidino-2-phenylindole-dihydrochloride
DCX: Doublecortin
ECM: Extracellular matrix
EF-2: Elongation factor 2
FA: Formic acid
FBS: Fetal bovine serum
FCS: Fetal calf serum
FDR: False discovery rate
FGF-2: Fibroblast growth factor 2
GDN: Glia-derived nexin
GFP: Green fluorescent protein
hBM-MSC: Human bone marrow mesenchymal stem cell
HGF: Hepatocyte growth factor
hMSC: Human mesenchymal stem cell
hNPC: Human telencephalon neural precursor cell
Hsp70: Heat shock protein 70 kDa
hWJ-MSC: Human Wharton Jelly mesenchymal stem cell
hWJ-MSC CM: Human Wharton Jelly mesenchymal stem cell conditioned media
IDA: Information-dependent acquisition
IGF-2: Insulin growth factor-2
IgG: Immunoglobulin G
LC/MS: Liquid chromatography-mass spectrometry
m/z: Mass-to-charge ratio
MAP-2: Microtubule associated protein-2
MIF: Macrophage migration inhibitory factor
MMTS: Methyl methathiosulfonate
MRP-1: Multidrug resistance-associated protein 1
MS/MS: Tandem mass spectrometry
NGF: Nerve growth factor
PBS: Phosphate-buffered saline
PEDF: Pigment epithelium-derived factor
Prx1: Peroxiredoxin 1
ROS: Reactive oxygen species
SDF-1α: Stem cell-derived factor 1 alpha
SWATH: Sequential Windowed data independent Acquisition of the Total High-resolution Mass Spectra
TCA: Trichloroacetic acid
TCEP: Tris (2-carboxyethyl)phosphine
TEAB: Triethylammonium bicarbonate buffer
TGF-β1: Transforming growth factor beta 1
UCHL1: Ubiquitin carboxy-terminal hydrolase L1
VEGF: Vascular endothelial growth factor
XIC: Extracted-ion chromatogram

## References

[1] Kan I, Barhum Y, Melamed E, Offen D (2011). Mesenchymal stem cells stimulate endogenous neurogenesis in the subventricular zone of adult mice. Stem Cell Rev..

[2] Lindvall O, Kokaia Z (2010). Stem cells in human neurodegenerative disorders—time for clinical translation?. J Clin Invest..

[3] Shihabuddin LS, Aubert I (2010). Stem cell transplantation for neurometabolic and neurodegenerative diseases. Neuropharmacology..

[4] Pittenger MF, Mackay AM, Beck SC, Jaiswal RK, Douglas R, Mosca JD (1999). Multilineage potential of adult human mesenchymal stem cells. Science..

[5] Zuk PA, Zhu M, Ashjian P, De Ugarte DA, Huang JI, Mizuno H (2002). Human adipose tissue is a source of multipotent stem cells. Mol Biol Cell..

[6] Wang HS, Hung SC, Peng ST, Huang CC, Wei HM, Guo YJ (2004). Mesenchymal stem cells in the Wharton's jelly of the human umbilical cord. Stem Cells..

[7] Sabapathy V, Sundaram B, MS V, Mankuzhy P, Kumar S (2014). Human Wharton's Jelly mesenchymal stem cells plasticity augments scar-free skin wound healing with hair growth. PloS One.

[8] Batsali AK, Kastrinaki MC, Papadaki HA, Pontikoglou C (2013). Mesenchymal stem cells derived from Wharton's Jelly of the umbilical cord: biological properties and emerging clinical applications. Curr Stem Cell Res Ther..

[9] Taghizadeh RR, Cetrulo KJ, Cetrulo CL (2011). Wharton's Jelly stem cells: future clinical applications. Placenta..

[10] Weiss ML, Troyer DL (2006). Stem cells in the umbilical cord. Stem Cell Rev..

[11] Sarugaser R, Ennis J, Stanford WL, Davies JE (2009). Isolation, propagation, and characterization of human umbilical cord perivascular cells (HUCPVCs). Methods Mol Biol..

[12] Baksh D, Yao R, Tuan RS (2007). Comparison of proliferative and multilineage differentiation potential of human mesenchymal stem cells derived from umbilical cord and bone marrow. Stem Cells..

[13] Ribeiro CA, Fraga JS, Graos M, Neves NM, Reis RL, Gimble JM (2012). The secretome of stem cells isolated from the adipose tissue and Wharton jelly acts differently on central nervous system derived cell populations. Stem Cell Res Ther..

[14] Teixeira FG, Carvalho MM, Sousa N, Salgado AJ (2013). Mesenchymal stem cells secretome: a new paradigm for central nervous system regeneration?. Cell Mol Life Sci..

[15] Weiss ML, Medicetty S, Bledsoe AR, Rachakatla RS, Choi M, Merchav S (2006). Human umbilical cord matrix stem cells: preliminary characterization and effect of transplantation in a rodent model of Parkinson's disease. Stem Cells..

[16] Teixeira FG, Carvalho MM, Neves-Carvalho A, Panchalingam KM, Behie LA, Pinto L, et al. Secretome of mesenchymal progenitors from the umbilical cord acts as modulator of neural/glial proliferation and differentiation. Stem Cell Rev Rep. 2015;11:288–97. 10.1007/s12015-014-9576-2 25420577

[17] Roemeling-van Rhijn M, Mensah FK, Korevaar SS, Leijs MJ, van Osch GJ, Ijzermans JN (2013). Effects of hypoxia on the immunomodulatory properties of adipose tissue-derived mesenchymal stem cells. Front Immunol..

[18] Caplan AI (2007). Adult mesenchymal stem cells for tissue engineering versus regenerative medicine. J Cell Physiol..

[19] Jung S, Panchalingam KM, Wuerth RD, Rosenberg L, Behie LA (2012). Large-scale production of human mesenchymal stem cells for clinical applications. Biotechnol Appl Biochem..

[20] Nekanti U, Dastidar S, Venugopal P, Totey S, Ta M (2010). Increased proliferation and analysis of differential gene expression in human Wharton's jelly-derived mesenchymal stromal cells under hypoxia. Int J Biol Sci..

[21] Cicione C, Muinos-Lopez E, Hermida-Gomez T, Fuentes-Boquete I, Diaz-Prado S, Blanco FJ (2013). Effects of severe hypoxia on bone marrow mesenchymal stem cells differentiation potential. Stem Cells Int..

[22] Das R, Jahr H, van Osch GJ, Farrell E (2010). The role of hypoxia in bone marrow-derived mesenchymal stem cells: considerations for regenerative medicine approaches. Tissue Eng Part B Rev..

[23] Chow DC, Wenning LA, Miller WM, Papoutsakis ET (2001). Modeling pO(2) distributions in the bone marrow hematopoietic compartment. I. Krogh's model. Biophys J..

[24] Bizzarri A, Koehler H, Cajlakovic M, Pasic A, Schaupp L, Klimant I (2006). Continuous oxygen monitoring in subcutaneous adipose tissue using microdialysis. Anal Chim Acta..

[25] Harrison JS, Rameshwar P, Chang V, Bandari P (2002). Oxygen saturation in the bone marrow of healthy volunteers. Blood..

[26] Fischer B, Bavister BD (1993). Oxygen tension in the oviduct and uterus of rhesus monkeys, hamsters and rabbits. J Reprod Fertil..

[27] Ma T, Grayson WL, Frohlich M, Vunjak-Novakovic G (2009). Hypoxia and stem cell-based engineering of mesenchymal tissues. Biotechnol Prog..

[28] Ward JP (1777). Oxygen sensors in context. Biochim Biophys Acta..

[29] Lavrentieva A, Majore I, Kasper C, Hass R (2010). Effects of hypoxic culture conditions on umbilical cord-derived human mesenchymal stem cells. Cell Commun Signal..

[30] Tsai CC, Chen YJ, Yew TL, Chen LL, Wang JY, Chiu CH (2011). Hypoxia inhibits senescence and maintains mesenchymal stem cell properties through down-regulation of E2A-p21 by HIF-TWIST. Blood..

[31] Lee SH, Lee JH, Yoo SY, Hur J, Kim HS, Kwon SM (2013). Hypoxia inhibits cellular senescence to restore the therapeutic potential of old human endothelial progenitor cells via the hypoxia-inducible factor-1alpha-TWIST-p21 axis. Arterioscler Thromb Vasc Biol..

[32] Weijers EM, Van Den Broek LJ, Waaijman T, Van Hinsbergh VW, Gibbs S, Koolwijk P (2011). The influence of hypoxia and fibrinogen variants on the expansion and differentiation of adipose tissue-derived mesenchymal stem cells. Tissue Eng Part A..

[33] Valorani MG, Montelatici E, Germani A, Biddle A, D'Alessandro D, Strollo R (2012). Pre-culturing human adipose tissue mesenchymal stem cells under hypoxia increases their adipogenic and osteogenic differentiation potentials. Cell Prolif..

[34] Chuang TJ, Lin KC, Chio CC, Wang CC, Chang CP, Kuo JR (2012). Effects of secretome obtained from normoxia-preconditioned human mesenchymal stem cells in traumatic brain injury rats. J Trauma Acute Care Surg..

[35] Chang CP, Chio CC, Cheong CU, Chao CM, Cheng BC, Lin MT (2013). Hypoxic preconditioning enhances the therapeutic potential of the secretome from cultured human mesenchymal stem cells in experimental traumatic brain injury. Clin Sci..

[36] Liu L, Gao J, Yuan Y, Chang Q, Liao Y, Lu F (2013). Hypoxia preconditioned human adipose derived mesenchymal stem cells enhance angiogenic potential via secretion of increased VEGF and bFGF. Cell Biology Int..

[37] Volkmer E, Drosse I, Otto S, Stangelmayer A, Stengele M, Kallukalam BC (2008). Hypoxia in static and dynamic 3D culture systems for tissue engineering of bone. Tissue Eng Part A..

[38] Yeatts AB, Choquette DT, Fisher JP (1830). Bioreactors to influence stem cell fate: augmentation of mesenchymal stem cell signaling pathways via dynamic culture systems. Biochim Biophys Acta..

[39] Santos F, Andrade PZ, Abecasis MM, Gimble JM, Chase LG, Campbell AM (2011). Toward a clinical-grade expansion of mesenchymal stem cells from human sources: a microcarrier-based culture system under xeno-free conditions. Tissue Eng Part C Methods..

[40] Hewitt CJ, Lee K, Nienow AW, Thomas RJ, Smith M, Thomas CR (2011). Expansion of human mesenchymal stem cells on microcarriers. Biotechnol Lett..

[41] Jung S, Sen A, Rosenberg L, Behie LA (2012). Human mesenchymal stem cell culture: rapid and efficient isolation and expansion in a defined serum-free medium. J Tissue Eng Regen Med..

[42] Baghbaderani BA, Mukhida K, Sen A, Kallos MS, Hong M, Mendez I (2010). Bioreactor expansion of human neural precursor cells in serum-free media retains neurogenic potential. Biotechnol Bioeng..

[43] Mendez I, Sanchez-Pernaute R, Cooper O, Vinuela A, Ferrari D, Bjorklund L (2005). Cell type analysis of functional fetal dopamine cell suspension transplants in the striatum and substantia nigra of patients with Parkinson's disease. Brain..

[44] Mendez I, Dagher A, Hong M, Gaudet P, Weerasinghe S, McAlister V (2002). Simultaneous intrastriatal and intranigral fetal dopaminergic grafts in patients with Parkinson disease: a pilot study. Report of three cases. J Neurosurg.

[45] Fraga JS, Silva NA, Lourenco AS, Goncalves V, Neves NM, Reis RL (2013). Unveiling the effects of the secretome of mesenchymal progenitors from the umbilical cord in different neuronal cell populations. Biochimie..

[46] Salgado AJ, Fraga JS, Mesquita AR, Neves NM, Reis RL, Sousa N (2010). Role of human umbilical cord mesenchymal progenitors conditioned media in neuronal/glial cell densities, viability, and proliferation. Stem Cells Dev..

[47] Manadas BJ, Vougas K, Fountoulakis M, Duarte CB (2006). Sample sonication after trichloroacetic acid precipitation increases protein recovery from cultured hippocampal neurons, and improves resolution and reproducibility in two-dimensional gel electrophoresis. Electrophoresis..

[48] Anjo SI, Santa C, Manadas B (2014). Short GeLC-SWATH: a fast and reliable quantitative approach for proteomic screenings. Proteomics..

[49] Tang WH, Shilov IV, Seymour SL (2008). Nonlinear fitting method for determining local false discovery rates from decoy database searches. J Proteome Res..

[50] Sennels L, Bukowski-Wills JC, Rappsilber J (2009). Improved results in proteomics by use of local and peptide-class specific false discovery rates. BMC Bioinformatics..

[51] Gillet LC, Navarro P, Tate S, Röst H, Selevsek N, Reiter L (2012). Targeted data extraction of the MS/MS spectra generated by data-independent acquisition: a new concept for consistent and accurate proteome analysis. Mol Cell Proteomics..

[52] Lambert J-P, Ivosev G, Couzens AL, Larsen B, Taipale M, Lin Z-Y (2013). Mapping differential interactomes by affinity purification coupled with data-independent mass spectrometry acquisition. Nat Methods..

[53] Collins BC, Gillet LC, Rosenberger G, Röst HL, Vichalkovski A, Gstaiger M (2013). Quantifying protein interaction dynamics by SWATH mass spectrometry: application to the 14-3-3 system. Nat Methods..

[54] Jung S, Sen A, Rosenberg L, Behie LA (2010). Identification of growth and attachment factors for the serum-free isolation and expansion of human mesenchymal stromal cells. Cytotherapy..

[55] Saller MM, Prall WC, Docheva D, Schonitzer V, Popov T, Anz D (2012). Increased stemness and migration of human mesenchymal stem cells in hypoxia is associated with altered integrin expression. Biochem Biophys Res Commun..

[56] Grant JL, Smith B (1963). Bone marrow gas tensions, bone marrow blood flow, and erythropoiesis in man. Ann Intern Med..

[57] Holzwarth C, Vaegler M, Gieseke F, Pfister SM, Handgretinger R, Kerst G (2010). Low physiologic oxygen tensions reduce proliferation and differentiation of human multipotent mesenchymal stromal cells. BMC Cell Biol..

[58] Basciano L, Nemos C, Foliguet B, de Isla N, de Carvalho M, Tran N (2011). Long term culture of mesenchymal stem cells in hypoxia promotes a genetic program maintaining their undifferentiated and multipotent status. BMC Cell Biol..

[59] Sart S, Liu Y, Ma T, Li Y (2014). Microenvironment regulation of pluripotent stem cell-derived neural progenitor aggregates by human mesenchymal stem cell secretome. Tissue Eng Part A..

[60] Park SM, Jung JS, Jang MS, Kang KS, Kang SK (2008). Transforming growth factor-beta1 regulates the fate of cultured spinal cord-derived neural progenitor cells. Cell Prolif..

[61] Liang W, Han Q, Jin W, Xiao Z, Huang J, Ni H (2010). The promotion of neurological recovery in the rat spinal cord crushed injury model by collagen-binding BDNF. Biomaterials..

[62] Wang F, Yasuhara T, Shingo T, Kameda M, Tajiri N, Yuan WJ (2010). Intravenous administration of mesenchymal stem cells exerts therapeutic effects on parkinsonian model of rats: focusing on neuroprotective effects of stromal cell-derived factor-1alpha. BMC Neurosci..

[63] Li QM, Fu YM, Shan ZY, Shen JL, Zhang XM, Lei L (2009). MSCs guide neurite directional extension and promote oligodendrogenesis in NSCs. Biochem Biophys Res Commun..

[64] Wang Y, Tu W, Lou Y, Xie A, Lai X, Guo F (2009). Mesenchymal stem cells regulate the proliferation and differentiation of neural stem cells through Notch signaling. Cell Biol Int..

[65] Farmer L, Sommer J, Monard D (1990). Glia-derived nexin potentiates neurite extension in hippocampal pyramidal cells in vitro. Dev Neurosci..

[66] Tizon B, Sahoo S, Yu H, Gauthier S, Kumar AR, Mohan P (2010). Induction of autophagy by cystatin C: a mechanism that protects murine primary cortical neurons and neuronal cell lines. PloS One..

[67] Sakurai M, Ayukawa K, Setsuie R, Nishikawa K, Hara Y, Ohashi H (2006). Ubiquitin C-terminal hydrolase L1 regulates the morphology of neural progenitor cells and modulates their differentiation. J Cell Sci..

[68] Zhu H, Santo A, Li Y (2012). The antioxidant enzyme peroxiredoxin and its protective role in neurological disorders. Exp Biol Med..

[69] Chen J, Lee CT, Errico SL, Becker KG, Freed WJ (2007). Increases in expression of 14-3-3 eta and 14-3-3 zeta transcripts during neuroprotection induced by delta9-tetrahydrocannabinol in AF5 cells. J Neurosci Res..

[70] Pires Neto MA, Braga-de-Souza S, Lent R (1999). Extracellular matrix molecules play diverse roles in the growth and guidance of central nervous system axons. Braz J Med Biol Res..

[71] Sun W, Kim H (2007). Neurotrophic roles of the beta-thymosins in the development and regeneration of the nervous system. Ann N Y Acad Sci..

[72] Iketani M, Iizuka A, Sengoku K, Kurihara Y, Nakamura F, Sasaki Y (2013). Regulation of neurite outgrowth mediated by localized phosphorylation of protein translational factor eEF2 in growth cones. Dev Neurobiol..

[73] van Kesteren RE, Carter C, Dissel HM, van Minnen J, Gouwenberg Y, Syed NI (2006). Local synthesis of actin-binding protein beta-thymosin regulates neurite outgrowth. J Neurosci..

[74] Johnson G, Gotlib J, Haroutunian V, Bierer L, Nairn AC, Merril C (1992). Increased phosphorylation of elongation factor 2 in Alzheimer's disease. Brain Res Mol Brain Res..

[75] Hoffmann MC, Nitsch C, Scotti AL, Reinhard E, Monard D (1992). The prolonged presence of glia-derived nexin, an endogenous protease inhibitor, in the hippocampus after ischemia-induced delayed neuronal death. Neuroscience..

[76] Ghidoni R, Paterlini A, Albertini V, Glionna M, Monti E, Schiaffonati L (2011). Cystatin C is released in association with exosomes: a new tool of neuronal communication which is unbalanced in Alzheimer's disease. Neurobiol Aging..

[77] Gauthier S, Kaur G, Mi W, Tizon B, Levy E (2011). Protective mechanisms by cystatin C in neurodegenerative diseases. Front Biosci..

[78] D'Adamio L (2010). Role of cystatin C in neuroprotection and its therapeutic implications. Am J Pathol..

[79] Gong B, Leznik E (2007). The role of ubiquitin C-terminal hydrolase L1 in neurodegenerative disorders. Drug News Perspect..

[80] Kang SW, Shin YJ, Shim YJ, Jeong SY, Park IS, Min BH (2005). Clusterin interacts with SCLIP (SCG10-like protein) and promotes neurite outgrowth of PC12 cells. Exp Cell Res..

[81] Wicher G, Fex-Svenningsen A, Velsecchi I, Charnay Y, Aldskogius H (2008). Extracellular clusterin promotes neuronal network complexity in vitro. Neuroreport..

[82] Pucci S, Mazzarelli P, Missiroli F, Regine F, Ricci F (2008). Neuroprotection: VEGF, IL-6, and clusterin: the dark side of the moon. Prog Brain Res..

[83] Cimini A, Gentile R, Angelucci F, Benedetti E, Pitari G, Giordano A (2013). Neuroprotective effects of PrxI over-expression in an in vitro human Alzheimer's disease model. J Cell Biochem..

[84] Ramser EM, Wolters G, Dityateva G, Dityatev A, Schachner M, Tilling T (2010). The 14-3-3zeta protein binds to the cell adhesion molecule L1, promotes L1 phosphorylation by CKII and influences L1-dependent neurite outgrowth. PloS One..

[85] Wechsler-Reya RJ (2001). Caught in the matrix: how vitronectin controls neuronal differentiation. Trends Neurosci..

[86] Pons S, Marti E (2000). Sonic hedgehog synergizes with the extracellular matrix protein vitronectin to induce spinal motor neuron differentiation. Development..

[87] Gil JE, Woo DH, Shim JH, Kim SE, You HJ, Park SH (2009). Vitronectin promotes oligodendrocyte differentiation during neurogenesis of human embryonic stem cells. FEBS Lett..

[88] Rush T, Liu X, Nowakowski AB, Petering DH, Lobner D (2012). Glutathione-mediated neuroprotection against methylmercury neurotoxicity in cortical culture is dependent on MRP1. Neurotoxicology..

[89] Park HA, Kubicki N, Gnyawali S, Chan YC, Roy S, Khanna S (2011). Natural vitamin E alpha-tocotrienol protects against ischemic stroke by induction of multidrug resistance-associated protein 1. Stroke..

[90] Crigler L, Robey RC, Asawachaicharn A, Gaupp D, Phinney DG (2006). Human mesenchymal stem cell subpopulations express a variety of neuro-regulatory molecules and promote neuronal cell survival and neuritogenesis. Exp Neurol..

[91] Yabe T, Sanagi T, Yamada H (2010). The neuroprotective role of PEDF: implication for the therapy of neurological disorders. Curr Mol Med..

[92] Ramirez-Castillejo C, Sanchez-Sanchez F, Andreu-Agullo C, Ferron SR, Aroca-Aguilar JD, Sanchez P (2006). Pigment epithelium-derived factor is a niche signal for neural stem cell renewal. Nat Neurosci..

[93] Mackay KB, Loddick SA, Naeve GS, Vana AM, Verge GM, Foster AC (2003). Neuroprotective effects of insulin-like growth factor-binding protein ligand inhibitors in vitro and in vivo. J Cereb Blood Flow Metab..

[94] Feldman EL, Sullivan KA, Kim B, Russell JW (1997). Insulin-like growth factors regulate neuronal differentiation and survival. Neurobiol Dis..

[95] Pasterkamp RJ, Peschon JJ, Spriggs MK, Kolodkin AL (2003). Semaphorin 7A promotes axon outgrowth through integrins and MAPKs. Nature..

[96] Pasterkamp RJ, Kolodkin AL (2003). Semaphorin junction: making tracks toward neural connectivity. Curr Opin Neurobiol..

[97] Kim JY, Kim N, Zheng Z, Lee JE, Yenari MA (2013). The 70 kDa heat shock protein protects against experimental traumatic brain injury. Neurobiol Dis..

[98] Antoine-Bertrand J, Ghogha A, Luangrath V, Bedford FK, Lamarche-Vane N (2011). The activation of ezrin-radixin-moesin proteins is regulated by netrin-1 through Src kinase and RhoA/Rho kinase activities and mediates netrin-1-induced axon outgrowth. Mol Biol Cell..

[99] Paglini G, Kunda P, Quiroga S, Kosik K, Caceres A (1998). Suppression of radixin and moesin alters growth cone morphology, motility, and process formation in primary cultured neurons. J Cell Biol..

[100] Hsiao ST, Lokmic Z, Peshavariya H, Abberton KM, Dusting GJ, Lim SY (2013). Hypoxic conditioning enhances the angiogenic paracrine activity of human adipose-derived stem cells. Stem Cells Dev..

[101] Ribeiro CA, Salgado AJ, Fraga JS, Silva NA, Reis RL, Sousa N (2011). The secretome of bone marrow mesenchymal stem cells-conditioned media varies with time and drives a distinct effect on mature neurons and glial cells (primary cultures). J Tissue Eng Regen Med..

[102] Dos Santos F, Andrade PZ, Boura JS, Abecasis MM, da Silva CL, Cabral JM (2010). Ex vivo expansion of human mesenchymal stem cells: a more effective cell proliferation kinetics and metabolism under hypoxia. J Cell Physiol..

[103] Ohnishi S, Yasuda T, Kitamura S, Nagaya N (2007). Effect of hypoxia on gene expression of bone marrow-derived mesenchymal stem cells and mononuclear cells. Stem Cells..

[104] Gui C, Wang JA, He AN, Chen TL, Luo RH, Jiang J (2007). Heregulin protects mesenchymal stem cells from serum deprivation and hypoxia-induced apoptosis. Mol Cell Biochem..

[105] Hung SC, Pochampally RR, Chen SC, Hsu SC, Prockop DJ (2007). Angiogenic effects of human multipotent stromal cell conditioned medium activate the PI3K-Akt pathway in hypoxic endothelial cells to inhibit apoptosis, increase survival, and stimulate angiogenesis. Stem Cells..

[106] Rosova I, Dao M, Capoccia B, Link D, Nolta JA (2008). Hypoxic preconditioning results in increased motility and improved therapeutic potential of human mesenchymal stem cells. Stem Cells..

[107] Hu X, Yu SP, Fraser JL, Lu Z, Ogle ME, Wang JA (2008). Transplantation of hypoxia-preconditioned mesenchymal stem cells improves infarcted heart function via enhanced survival of implanted cells and angiogenesis. J Thorac Cardiovasc Surg..

